# Seminal Simian Immunodeficiency Virus in Chronically Infected Cynomolgus Macaques Is Dominated by Virus Originating from Multiple Genital Organs

**DOI:** 10.1128/JVI.00133-18

**Published:** 2018-06-29

**Authors:** Laurent Houzet, Marcos Pérez-Losada, Giulia Matusali, Claire Deleage, Nathalie Dereuddre-Bosquet, Anne-Pascale Satie, Florence Aubry, Emmanuelle Becker, Bernard Jégou, Roger Le Grand, Brandon F. Keele, Keith A. Crandall, Nathalie Dejucq-Rainsford

**Affiliations:** a Université Rennes, Inserm, EHESP, Irset (Institut de recherche en santé, environnement et travail)—UMR_S 1085, Rennes, France; b Computational Biology Institute, Milken Institute School of Public Health, George Washington University, Ashburn, Virginia, USA; c CIBIO-InBIO, Universidade do Porto, Campus Agrário de Vairão, Vairão, Portugal; d CEA—Université Paris-Sud—INSERM U1184, Immunology of Viral Infections and Autoimmune Diseases, IDMIT Department, IBFJ, Fontenay-aux-Roses and Kremlin-Bicêtre, France; e AIDS and Cancer Virus Program, Leidos Biomedical Research, Inc., Frederick National Laboratory for Cancer Research, Frederick, Maryland, USA; Emory University

**Keywords:** virus, sexual transmission, semen, HIV, male genital organs, cynomolgus macaque, SIV, phylogenetic analysis, single-genome amplification, compartmentalization, simian immunodeficiency virus

## Abstract

The sexual transmission of viruses is responsible for the spread of multiple infectious diseases. Although the human immunodeficiency virus (HIV)/AIDS pandemic remains fueled by sexual contacts with infected semen, the origin of virus in semen is still unknown. In a substantial number of HIV-infected men, viral strains present in semen differ from the ones in blood, suggesting that HIV is locally produced within the genital tract. Such local production may be responsible for the persistence of HIV in semen despite effective antiretroviral therapy. In this study, we used single-genome amplification, amplicon sequencing (*env* gene), and phylogenetic analyses to compare the genetic structures of simian immunodeficiency virus (SIV) populations across all the male genital organs and blood in intravenously inoculated cynomolgus macaques in the chronic stage of infection. Examination of the virus populations present in the male genital tissues of the macaques revealed compartmentalized SIV populations in testis, epididymis, vas deferens, seminal vesicles, and urethra. We found genetic similarities between the viral strains present in semen and those in epididymis, vas deferens, and seminal vesicles. The contribution of male genital organs to virus shedding in semen varied among individuals and could not be predicted based on their infection or proinflammatory cytokine mRNA levels. These data indicate that rather than a single source, multiple genital organs are involved in the release of free virus and infected cells into semen. These findings have important implications for our understanding of systemic virus shedding and persistence in semen and for the design of eradication strategies to access viral reservoirs.

**IMPORTANCE** Semen is instrumental for the dissemination of viruses through sexual contacts. Worryingly, a number of systemic viruses, such as HIV, can persist in this body fluid in the absence of viremia. The local source(s) of virus in semen, however, remains unknown. To elucidate the anatomic origin(s) of the virus released in semen, we compared viral populations present in semen with those in the male genital organs and blood of the Asian macaque model, using single-genome amplification, amplicon sequencing (*env* gene), and phylogenetic analysis. Our results show that multiple genital tissues harbor compartmentalized strains, some of them (i.e., from epididymis, vas deferens, and seminal vesicles) displaying genetic similarities with the viral populations present in semen. This study is the first to uncover local genital sources of viral populations in semen, providing a new basis for innovative targeted strategies to prevent and eradicate HIV in the male genital tract.

## INTRODUCTION

Semen plays a key role in the sexual transmission of viruses. It is the foremost vector of dissemination of human immunodeficiency virus type 1 (HIV-1), the causative agent of the AIDS pandemic. About 37 million people are infected by HIV-1 worldwide, and every year an average of 2 million people become infected, most commonly through sexual contact. Although antiretroviral treatments drastically decrease HIV transmission, HIV RNA and infected cells persist in semen despite undetectable blood viral load in about 8% of men receiving suppressive antiretroviral therapy for more than 6 months, suggesting the existence of local sources of virus within the male genital tract. Indeed, multiple phylogenetic studies have demonstrated that HIV strains in semen are distinct from those in the blood (i.e., compartmentalized) in over 60% of HIV-infected individuals (reviewed in reference 1). Yet the local source(s) of HIV in semen has never been identified. Early studies based on viral load measurements suggested that seminal HIV originates from the prostate or urethra (2, 3), whereas the only phylogenetic attempt performed to date on genital organs together with semen failed to identify compartmentalized strains in infected macaques (4). Recent outbreaks of the emerging Ebola and Zika viruses showed viral infiltration of and persistence in semen for extended periods after the systemic resolution of the infection, leading to cases of sexual transmission from asymptomatic men (reviewed in references 5 and 6). These worrisome findings further highlight our gap of knowledge regarding the mechanisms of semen contamination by viruses. The identification of the organs and cells responsible for semen contamination is indeed paramount to the design of targeted eradication strategies to prevent sexual transmission of viruses.

Semen is the end product of excretions from multiple genital organs, which combine and travel through specific ducts. Our laboratory (7–10) and others (11–13) demonstrated that these organs and ducts (i.e., testes, epididymis, vas deferens, seminal vesicles, prostate, and urethra) are infected by HIV/simian immunodeficiency virus (SIV) and may, therefore, constitute a source of virus in semen (reviewed in reference 1). In this study, we sought to decipher the origin of the viral particles and infected cells present in semen. For this, we took advantage of the cynomolgus macaque model of HIV infection that we previously validated through the demonstration of a pattern of genital organ infection and viral release in semen similar to that in men (9, 14, 15). We first determined the viral loads and inflammatory levels of all of the four male genital organs (testis, epididymis, seminal vesicles, and prostate) and two ducts (vas deferens and urethra) which represent potential sources of virus in semen. We next compared the viral populations in seminal plasma and seminal cells along with all those male genital organs and ducts, as well as blood plasma and blood cells, using single-genome amplification (SGA) optimized for large screening (16), amplicon sequencing (*env* gene), and phylogenetic and population genetic analyses. Our results revealed that viral compartmentalization occurs in different male genital organs, indicative of sustained local replication. Most importantly, we uncovered for the first time several male genital organs involved in the release of viral particles and infected cells into semen. These findings highlight how virus replication within the male genital tract can influence virus populations in semen and impact virus transmission.

## RESULTS

### Study population.

We screened 8 adult cynomolgus macaques intravenously inoculated with SIVmac251, in the chronic stage of infection (6 to 15 months postinfection), displaying blood viral loads between 4 and 5 log copies/ml. Two animals had high (H) viral loads in seminal plasma, i.e., ≥10^5^ copies/ml (H1 [21362] and H2 [30855]), three had moderate (M) seminal viral loads, i.e., between 10^3^ and 5 × 10^4^ copies/ml (M1 [29860], M2 [10999], and M3 [30690]) and three had low (L) (1.4 × 10^2^ copies/ml) to undetectable viral loads (L1 [19554], L2 [30569], and L3 [29965]) (Table 1). To facilitate their identification, the animals are referred to here according to their semen viral load classification (H, M, and L) instead of identification (ID) number. In order to preserve the seminal cells for viral genetic analyses, the cell-associated viral loads in semen were not determined. The comparison of blood and semen viral loads showed no correlation (Spearman test, *r* = −0.25; *P* = 0.54); suggesting that, at least in some animals, the virus present in the semen could be from a local origin.

**TABLE 1:** Summary of study animals^*a*^

Parameter	Value for macaque							
	H1 (21362)	H2 (30855)	M1 (29860)	M2 (10999)	M3 (30690)	L1 (19554)	L2 (30569)	L3 (29965)
SIV infection (mo)	12	6	14	14	15	6	15	15
Blood VL	1.4 × 10^5^	4 × 10^4^	6.3 × 10^4^	1.2 × 10^5^	7.4 × 10^4^	2 × 10^4^	1.8 × 10^5^	2 × 10^5^
Semen VL	1 × 10^5^	6.3 × 10^5^	1 × 10^4^	1 × 10^3^	5 × 10^4^	ND	1.4 × 10^2^	ND
T cells/CD4^+^ (cells/μl)	45	409	521	795	1161	230	141	34

a VL, viral load; ND, not determined.

### Semen viral load does not correlate with a single genital organ's infection and cytokine mRNA levels.

**FIG 1:**
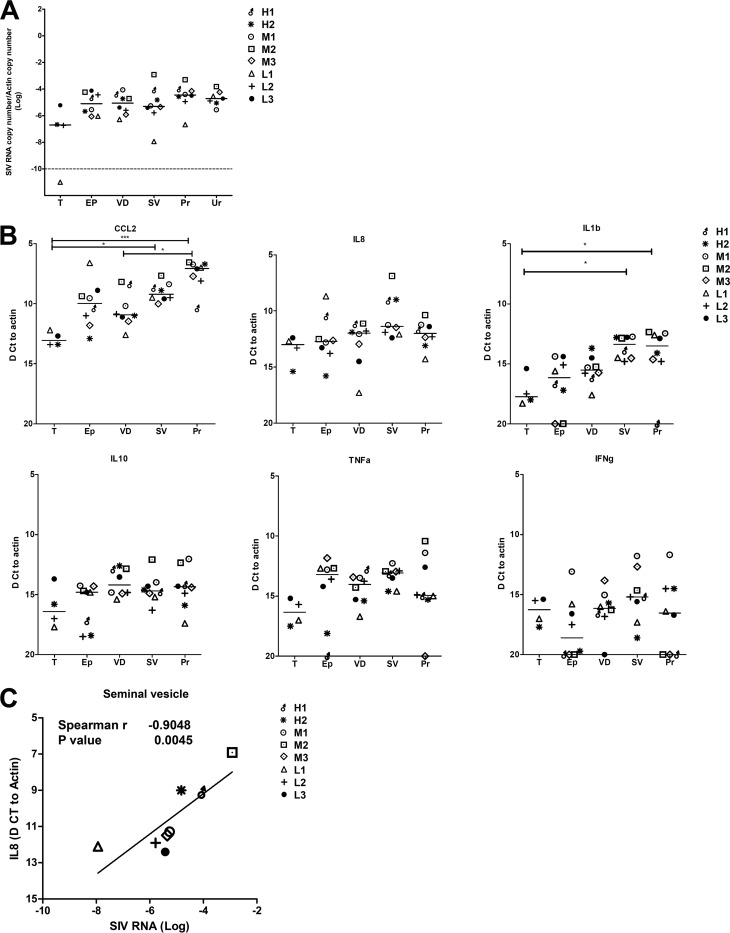
Infection and inflammation levels in male genital organs. SIV RNA (A) and cytokine mRNA (B) levels measured in the male genital organs (T, testis; EP, epididymis; VD, vas deferens; SV, seminal vesicles; Pr, prostate; UR, urethra) of the eight macaques of the study. Because of limited material, urethra had to be excluded from cytokine mRNA analysis. Statistical analysis was performed using the Mann-Whitney nonparametric test (*, *P* < 0.05; **, *P* < 0.01; ***, *P* < 0.001). (C) Correlation between SIV RNA and cytokine level in male genital organs. A correlation was observed with IL-8 in seminal vesicles.

We first sought to determine whether viral load in semen could be linked to infection/inflammation levels in any of the male genital organs responsible for semen production and transport. Using a sensitive two-step reverse transcription-quantitative PCR (RT-qPCR) for SIV *gag* RNA, we measured infection levels in testis, epididymis, vas deferens, seminal vesicle, prostate, and urethra (Fig. 1A). Viral RNA (vRNA) was detected in all the genital tissues analyzed, the lowest level being found in the testis and highest in the prostate. The comparison of vRNA levels in organs with semen and blood viral loads did not reveal any correlation and therefore could not be used to discriminate potential sources of semen viral load among those organs (Table 2).

**TABLE 2:** Statistical analysis of correlation between SIV RNA in genital organs and blood and semen viral loads

Organ	Spearman rho (*P* value^*a*^)	
	Blood viral load	Semen viral load
Testis	0.80 (0.333)	0.11 (0.917)
Epididymis	0.79 (0.028)	−0.38 (0.360)
Vas deferens	0.14 (0.752)	0.47 (0.243)
Seminal vesicles	0.05 (0.935)	0.67 (0.083)
Prostate	0.29 (0.501)	0.42 (0.299)

a Cutoff for statistical significance, *P* ≤ 0.01 (Bonferroni correction for multiple comparisons).

Because viral load in semen has been correlated in several studies to the seminal concentrations of various inflammatory cytokines (17–21), we also tested whether inflammation levels in male genital organs could influence tissue viral load and shedding in semen. Using mRNAs of 6 cytokines, which are known to correlate with HIV semen levels (tumor necrosis factor alpha [TNF-α], interleukin 1β [IL-1β], gamma interferon [IFN-γ], CCL2, and IL-10) (18, 19, 21, 22) or are elevated in HIV-infected patients (IL-8) (20, 23, 24), we measured inflammation levels within all the male genital organs, excluding the urethra due to limited material (Fig. 1B). Interestingly, CCL2 and IL-1β levels varied significantly among organs; however, no correlations were found between cytokine levels in genital tissues and semen viral load (Table 3). After correction for multiple comparisons, a single significant correlation was found between IL-8 mRNA and HIV RNA levels within the seminal vesicles (Spearman test, *r* = 0.90; *P* = 0.005) (Table 4 and Fig. 1C). In summary, we found that a wide range of male genital organs are infected by SIV and that neither vRNA levels nor the mRNA levels of the cytokines tested within those organs can be used to predict the release of SIV in semen.

**TABLE 3:** Statistical analysis of correlation between semen plasma viral load and individual cytokine level within each genital organ

Organ	Spearman rho (*P* value^*a*^)					
	CCL2	IL-8	IL-1β	IL-10	TNF-α	IFN-γ
Testis	0.89 (0.083)	0.95 (0.083)	0.11 (0.917)	−0.11 (0.917)	0.63 (0.417)	0.63 (0.417)
Epididymis	0.80 (0.022)	0.20 (0.619)	0.55 (0.297)	0.06 (0.882)	−0.02 (0.964)	0.36 (0.517)
Vas deferens	−0.34 (0.428)	−0.47 (0.243)	−0.24 (0.582)	−0.51 (0.197)	−0.46 (0.268)	−0.32 (0.498)
Seminal vesicles	−0.28 (0.501)	−0.73 (0.046)	−0.17 (0.665)	−0.14 (0.752)	0.01 (0.977)	−0.11 (0.793)
Prostate	−0.01 (0.977)	0.08 (0.840)	0.16 (0.713)	−0.14 (0.752)	0.32 (0.498)	−0.79 (0.133)

a Cutoff for statistical significance, *P* ≤ 0.008 (Bonferroni correction for multiple comparisons).

**TABLE 4:** Statistical analysis of correlation between SIV RNA and cytokine levels within the genital organs

Organ	Spearman rho (*P* value^*a*^)					
	CCL2	IL-8	IL-1β	IL-10	TNF-α	IFN-γ
Testis	0.32 (0.750)	−0.20 (0.917)	−0.80 (0.333)	−1.00 (0.083)	−0.40 (0.750)	−0.40 (0.750)
Epididymis	−0.36 (0.389)	0.21 (0.619)	−0.43 (0.419)	0.22 (0.619)	0.39 (0.396)	0.10 (0.950)
Vas deferens	−0.83 (0.015)	−0.60 (0.132)	−0.33 (0.428)	−0.61 (0.105)	−0.43 (0.299)	0.07 (0.906)
Seminal vesicles	−0.71 (0.058)	**−0.90 (0.005)**	−0.42 (0.299)	−0.69 (0.069)	−0.18 (0.665)	−0.28 (0.501)
Prostate	−0.07 (0.882)	−0.69 (0.069)	−0.39 (0.396)	−0.79 (0.028)	−0.56 (0.200)	−0.36 (0.517)

a A *P* value of ≤0.008 is considered statistically significant after Bonferroni correction for multiple comparisons. Significant values are shown in bold.

### Semen viral populations are distinct from blood viral populations in animals with high or moderate semen viral loads.

**FIG 2:**
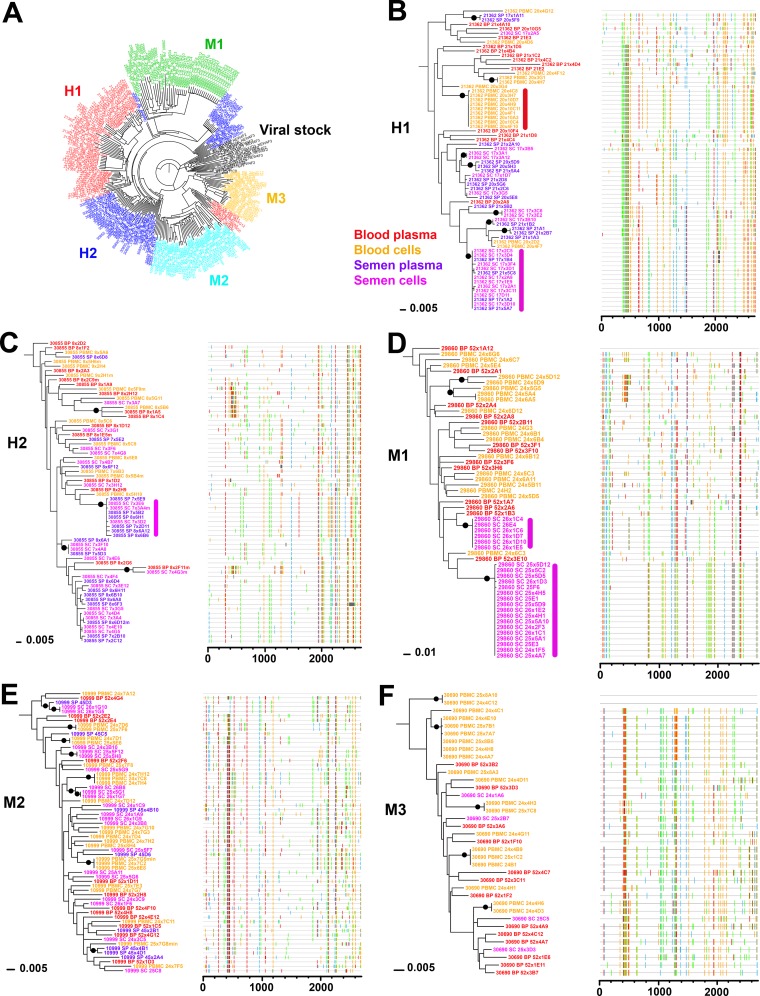
Phylogenetic analysis of blood and semen sequences. (A) Maximum likelihood phylogenetic tree generated from all subject sequences (blood and semen) together with viral stock demonstrating absence of cross-contamination (H1, red; H2, dark blue; M1, green; M2, light blue; M3, orange; viral stock, black). (B to F) Maximum likelihood phylogenetic trees (left) and Highlighter plots (right) generated from sequences derived from blood (peripheral blood mononuclear cells, orange; blood plasma, red) and semen (semen cells, purple; semen plasma, pink) of macaques H1 (B), H2 (C), M1 (D), M2 (E), and M3 (F). A clonal amplification-like profile is indicated by a solid bar. Filled circles indicate branches with bootstrap values of ≥70%. The tree was rooted using the viral inoculum sequences (not shown for visual clarity).

Genetic divergence between blood and semen virus populations has been shown to occur in about 60% of the over 100 HIV-infected men analyzed in multiple studies (reviewed in reference 1). Thus, we sought to identify in the macaques' semen a specific genetic profile that could be used as a signature to track the origins of the virus. In the 3 animals displaying low to undetectable viral loads in seminal plasma (L1, L2, and L3), no viral amplicon could be rescued from either seminal plasma or seminal cells. Among the 3 animals with moderate seminal viral loads, two had viral amplicons in seminal cells but not in seminal plasma (M1 and M3), while one had amplicons in both seminal cells and plasma (M2). In the 2 animals with high seminal viral loads (H1 and H2), viral amplicons were generated from both seminal cells and seminal plasma. The number of amplicons is presented in Table 5. The distinct viral populations found in those animals demonstrated the absence of laboratory cross-contamination (Fig. 2A).

**TABLE 5:** Analysis of SIV population inference in blood, semen, and viral stock^*a*^

Macaque (sequence)	Compartment	No. of sequences	Genetic diversity (DNAsp)		Population recombination rate (LDhat), rho^*d*^	Molecular adaptation (HyPhy), ω^*e*^/sites	Stop codons,N/h^*f*^
			Pi^*b*^	Theta^*c*^			
H1 (2,694 bp)	BP	13	0.014	0.016	100	0.424/2	0
	PBMC	17	0.009	0.01	17	0.537/3	1
	SP	19	0.009	0.01	10	0.529/2	0
	SC	19	0.007	0.009	6	0.471/2	1
H2 (2,658 bp)	BP	14	0.013	0.016	100	0.663/4	0
	PBMC	13	0.011	0.014	100	0.429/5	0
	SP	19	0.008	0.009	13	0.509/0	0
	SC	20	0.01	0.014	25	0.620/0	1
M1 (2,688 bp)	BP	13	0.011	0.014	100	0.317/2	0
	PBMC	19	0.013	0.015	100	0.376/5	0
	SP	0	NA	NA	NA	NA	NA
	SC	23	0.006	0.004	0	0.335/0	7 (4)
M2 (2,679 bp)	BP	12	0.013	0.015	100	0.522/2	1 (3)
	PBMC	23	0.011	0.014	63	0.531/4	0
	SP	8	0.013	0.014	100	0.49/1	1 (1)
	SC	20	0.011	0.014	100	0.35/2	1 (1)
M3 (2,649 bp)	BP	13	0.013	0.015	100	0.74/4	1 (1)
	PBMC	20	0.013	0.012	16	0.976/7	1 (1)
	SP	0	NA	NA	NA	NA	NA
	SC	4	0.016	0.015	4	0.837/4	0
Viral stock (2,690 bp)	ST	21	0.015	0.019	100	0.701/0	1 (2)

a BP, blood plasma; PBMC, peripheral blood mononuclear cells; SP, seminal plasma; SC, seminal cells; NA, not available.

b Nucleotide diversity (pi) = the average number of nucleotide differences per site between two sequences.

c Genetic diversity (theta) = 2Ne/mu, where Ne is the effective population size and mu is the per-generation mutation rate of the population of interest.

d Population recombination rate (rho) = 2Ner, where Ne is the effective population size and r is the genetic map distance across the region analyzed (the product of the physical distance and the per-site rate of recombination across the region).

e Natural selection (omega) = *dN/dS* (ratio of nonsynonymous [amino acid altering]/synonymous [silent] substitutions). Omega measures the selective pressure at the protein level, with an omega value of 1 meaning neutral mutations (no selection), an omega value of <1 meaning purifying (negative) selection, and an omega value of >1 meaning diversifying positive selection.

f Number of total stop codons (N) per number of haplotypes with stop codons (h).

We applied Wright's fixation index of population subdivision (*F*_st_) to evaluate virus compartmentalization between blood and semen in the five animals in which full-length *env* was amplified from semen (H1, H2, M1, M2, and M3). *F*_st_ is a measure of population differentiation due to genetic structure, with values ranging from 0 (absence of compartmentalization) to 1 (complete compartmentalization). Three additional methods (association index [AI], Slatkin-Maddison index [SM], and correlation coefficients *r* [correlation coefficient by length of branches] and *r_b_* [correlation coefficient by number of branches]) were used to validate compartmentalization. Viral populations were considered compartmentalized when all four tests converged. Semen/blood compartmentalization was statistically significant in 2/2 animals with high viral loads (H1 and H2) and in 1/3 animals with moderate viral loads (M1) and with a low to medium genetic structuring level (*F*_st_ range, 0.13 to 0.36) (Table 6). The genetic segregation of virus between semen and blood in these 3 macaques was confirmed by the topology of the corresponding maximum likelihood phylogenetic trees showing that semen and blood variants formed separate main clusters (Fig. 2B to D). Interestingly, highlighted plots showed that most of these clusters were associated with specific sequence motifs, which were absent or poorly represented in blood (Fig. 2B to D). In contrast, semen and blood viral populations were equilibrated, showing no evidence of compartmentalization in two macaques with moderate viral loads (M2 and M3), as indicated by compartmentalization analyses (Table 6). In agreement with this result, phylogenetic trees showed equilibrated viral populations with closely related clusters containing intermixed blood and semen sequences (Fig. 2E and F). Of note, no evidence of compartmentalization was ever observed between free virus particles in seminal plasma and infected seminal cells in any animal analyzed (Table 6). Population genetic analyses revealed that semen virus populations in animals H1, H2, and M1 showed lower genetic diversity (pi and theta) and recombination (rho) rates than blood virus populations (Table 5). Natural selection estimates (ω/sites) were similar across blood and semen, suggesting that all viral populations experienced similar degrees of selection pressure from the host immune system. Additionally, the frequency of internal stop codons in the *env* gene was very low in all samples, indicating the targeting of actively replicating viral populations (Table 5). In summary, 3 macaques (H1, H2, and M1) with high or moderate seminal viral loads above 3 logs showed compartmentalization between blood and semen virus populations, resulting in population-specific genetic profiles that could be used for virus tracking among genital organs.

**TABLE 6:** Genetic distance between blood and semen virus populations^*a*^

Macaque	*F*_st_ for:				
	Seminal plasma vs indicated compartment		Seminal cells vs indicated compartment		Seminal cells vs seminal plasma
	Blood plasma	Blood cells	Blood plasma	Blood cells	
					
H1	**0.217**	**0.237**	**0.258**	**0.320**	0.034
H2	**0.216**	**0.243**	**0.131**	**0.159**	−0.005
M1	NA	NA	**0.352**	**0.338**	NA
M2	−0.009	0.019	0.034	0.024	0.027
M3	NA	NA	−0.059	0.082	NA

a Values in bold are significant after Bonferroni multiple test correction (H1, *P* = 0.00065; H2, *P* = 0.0007; M1, *P* = 0.0008). NA, not available because no amplicons were isolated from seminal plasma of macaques M1 and M3.

### Viral populations in male genital organs show variable levels of compartmentalization.

**FIG 3:**
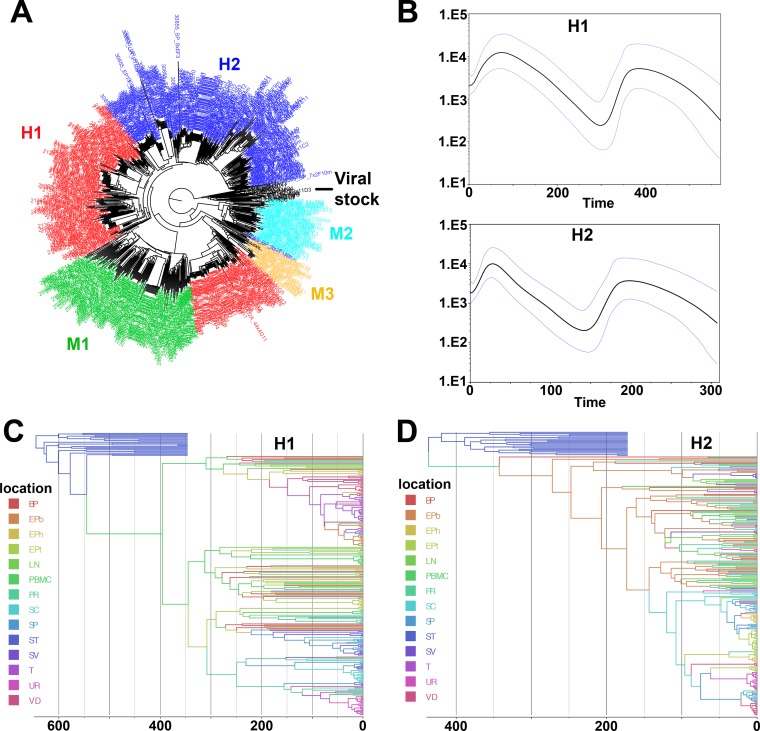
Population dynamic analysis of blood and genital tract virus populations derived from macaques H1 and H2. (A) Maximum likelihood phylogenetic tree generated from all macaque sequences (blood, semen, and male genital organs) together with viral stock demonstrating the absence of cross-contamination (H1, red; H2, dark blue; M1, green; M2, light blue; M3, orange; viral stock, black). (B) GMRF skyride plots generated from all sequences of macaques H1 (top) and H2 (bottom). (C and D) Bayesian maximum clade credibility tree generated from all sequences of macaques H1 (C) and H2 (D).

We next examined the viral populations present in testis, epididymis, vas deferens, seminal vesicle, prostate, and urethra of the three macaques (H1, H2, and M1) harboring a specific virus genotype in semen. Overall, SGA and amplicon sequencing yielded a total of 429 *env* sequences across the 3 macaques analyzed (Table 7) with no cross-contamination between samples (Fig. 3A). All male genital tract (MGT) tissues displayed similar numbers of stop codons, with no more than 10% of the sequences containing stop codons (Table 7). Except for urethra, which showed low genetic diversity and recombination rate, genetic diversity and population recombination varied greatly across body sites in macaques H1 and H2 but less in macaque M1 (Table 7). Similarly to the case with semen, there was no positive selection of viral populations in male genital organs. The dynamic population analysis of SIV in macaques H1 and H2 showed a bottleneck at the time of infection and then exponential growth with a small decline toward the end (Fig. 3B). Additionally, the Bayesian maximum clade credibility tree generated from all sequences of macaques H1 and H2 and viral inoculum suggests that infection initiated from a single variant (most recent common ancestor [MRCA]) for each macaque (Fig. 3C and D).

**TABLE 7:** Analysis of SIV population inference in organs

Macaque (sequence)	Tissue^*a*^	No. of sequences	Genetic diversity (DNAsp)		Population recombination rate (LDhat), rho	Molecular adaptation (HyPhy), ω/sites	Stop codons, N/h
			Pi	Theta			
H1 (2,694 bp)	T	21	0.002	0.005	0	0.648/0	1
	EPh	18	0.011	0.011	9	0.484/1	0
	EPb	17	0.003	0.005	5	0.927/0	0
	EPt	18	0.014	0.014	42	0.38/2	0
	VD	25	0.008	0.011	26	0.498/3	0
	SV	20	0.01	0.013	25	0.519/3	1
	PR	21	0.013	0.014	26	0.588/4	0
	UR	22	0.005	0.006	0	0.499/1	3 (1)
	LN	7	0.014	0.014	100	0.530/2	1
H2 (2,658 bp)	T	1	NA	NA	NA	NA	NA
	EPh	17	0.005	0.004	0	0.571/0	1
	EPb	20	0.01	0.013	100	0.466/7	0
	EPt	27	0.005	0.006	2	0.417/0	0
	VD	17	0.006	0.008	3	0.613/0	4 (2)
	SV	19	0.01	0.009	17	0.487/2	1
	PR	19	0.01	0.014	100	0.534/3	0
	UR	22	0.007	0.007	5	0.522/1	0
	LN	18	0.011	0.012	98	0.479/4	0
M1 (2,688 bp)	T	1	NA	NA	NA	NA	NA
	EPh	8	0.012	0.013	21	0.354/2	0
	EPb	12	0.012	0.013	24	0.336/1	0
	EPt	10	0.011	0.013	53	0.324/2	0
	VD	16	0.012	0.015	100	0.35/3	0
	SV	16	0.013	0.014	100	0.310/3	0
	PR	12	0.012	0.014	11	0.392/0	0
	UR	10	0.01	0.011	4	0.375/2	0
	LN	15	0.012	0.015	100	0.331/3	0

a T, testis; EPb, epididymis body; EPh, epididymis head; EPt, epididymis tail; VD, vas deferens; SV, seminal vesicles; PR, prostate; UR, urethra; LN, lymph node.

**FIG 4:**
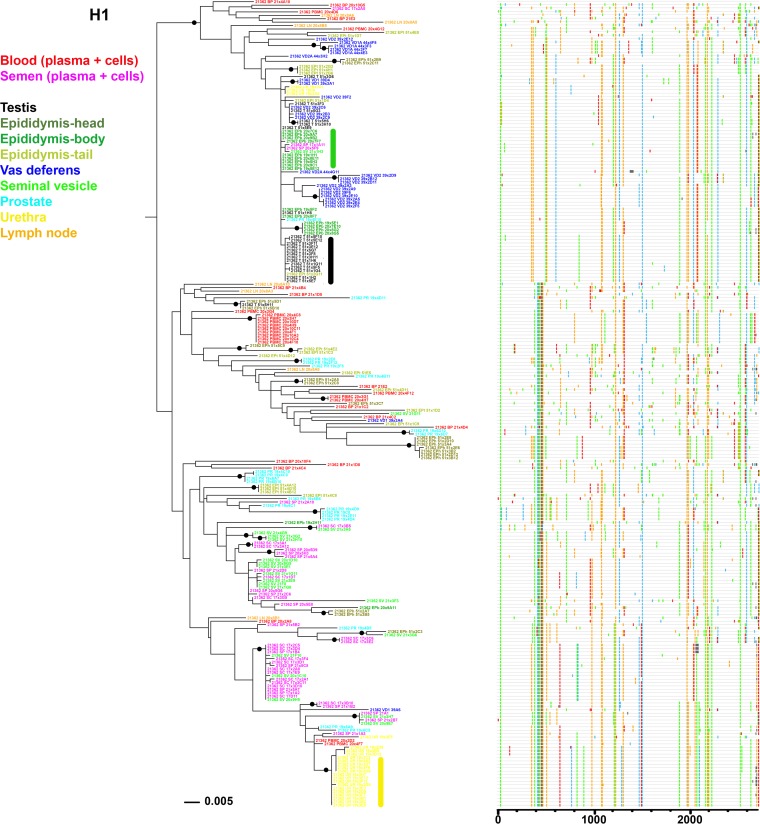
Analysis of SIV *env* sequences derived from blood, semen, and male genital organs from macaque H1. Shown are a maximum likelihood phylogenetic tree (left) and Highlighter plot (right) of SGA sequences derived from RNA samples. A clonal amplification-like profile is indicated by a solid bar. Filled circles indicate branches with bootstrap values greater than 70%. The tree was rooted using the viral inoculum sequences (not shown for visual clarity).

**FIG 5:**
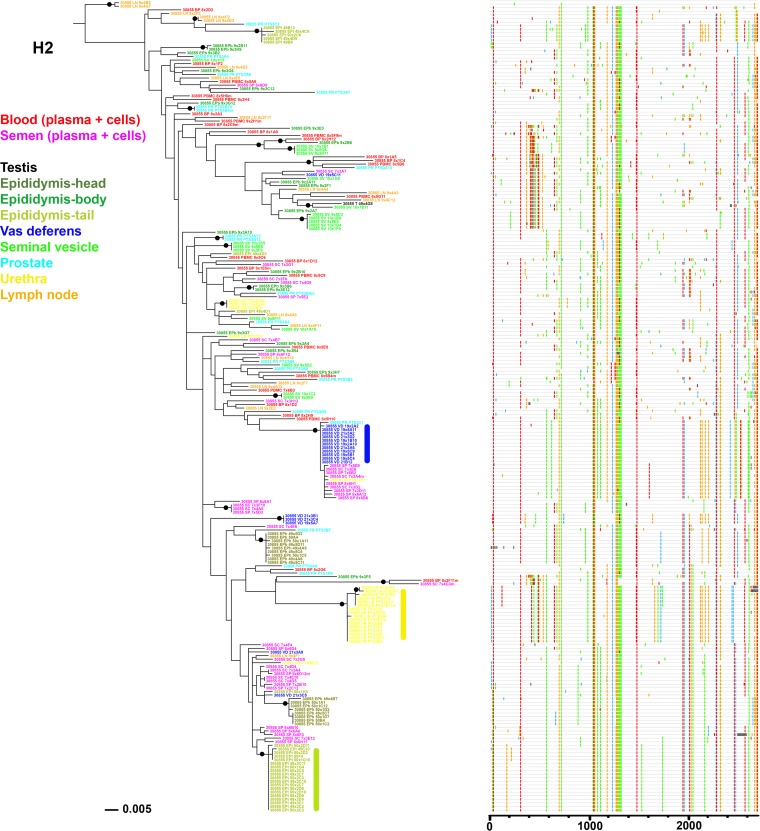
Analysis of SIV *env* sequences derived from blood, semen, and male genital organs from macaques H2. Shown is a maximum likelihood phylogenetic tree (left) and Highlighter plot (right) of SGA sequences derived from RNA samples. A clonal amplification-like profile is indicated by a solid bar. Filled circles indicate branches with bootstrap values of ≥70%. The tree was rooted using the viral inoculum sequences (not shown for visual clarity).

**FIG 6:**
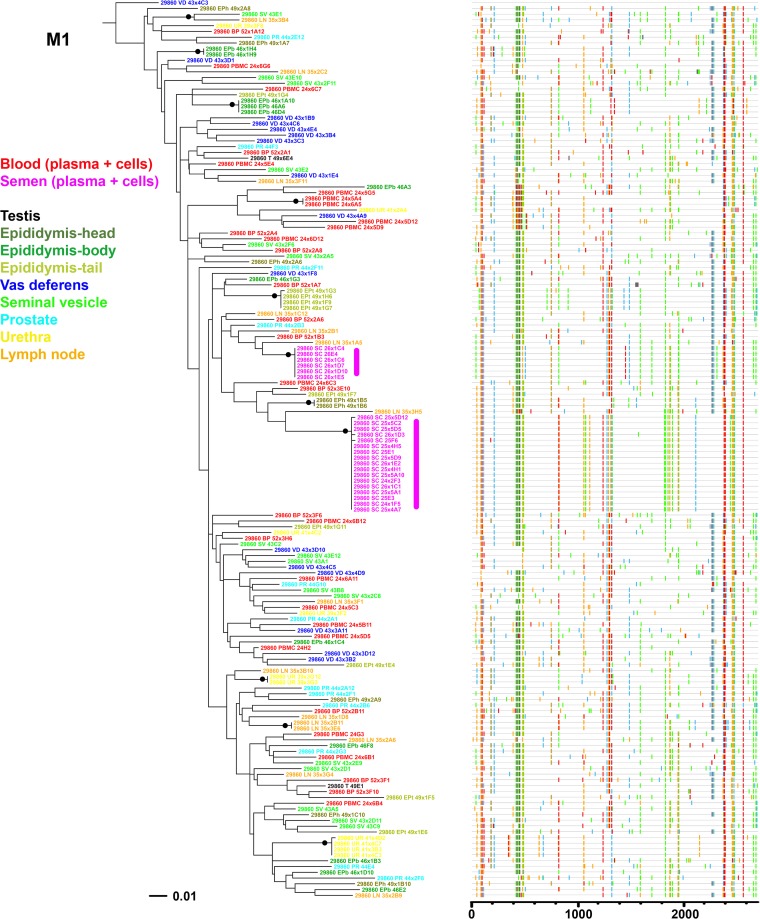
Analysis of SIV *env* sequences derived from blood, semen, and male genital organs from macaque M1. Shown is a maximum likelihood phylogenetic tree (left) and Highlighter plot (right) of SGA sequences derived from RNA samples. A clonal amplification-like profile is indicated by a solid bar. Filled circles indicate branches with bootstrap values of ≥70%. The tree was rooted using the viral inoculum sequences (not shown for visual clarity).

Organ compartmentalization was assessed to uncover tissue-specific viral populations. In macaque H1, significant evidence of isolation between organ and blood virus was observed for all 6 male genital organs, with higher levels of population structuring associated with testis, epididymis body, vas deferens, and urethra (range, 0.41 to 0.63) and lower levels associated with seminal vesicles and prostate (range, 0.11 to 0.25) (Table 8). Within the epididymis, the head and tail displayed significant differences in structuring with blood cells only and at a very low rate. In animal H2, medium to high levels of structuring (range, 0.30 to 0.42) were observed between blood (plasma and cells) and organs for the epididymis head and tail, vas deferens, and urethra. In contrast, in animal M1, only 2 genital organs (epididymis tail and urethra) showed low but significant levels of differentiation with blood virus (0.10 and 0.11, respectively). No differentiation between blood and inguinal lymph node viruses was ever observed. These results are in line with the phylogenetic trees and Highlighter plots showing that in macaques H1 and H2, most of the sequences from genital tissues with high compartmentalization levels were located within clusters separated from blood, harboring a population-specific phylogenetic profile (Fig. 4 and 5). In contrast, in animal M1, viral sequences from genital organs were intermingled with blood sequences (Fig. 6), although semen sequence clusters were separate. Compartmentalization in male genital organs was also associated with a significant loss in viral diversity and recombination rate, but no change in stop codon frequency (Table 7). Overall, these results demonstrate that genital organs can all display virus compartmentalization to various degrees, highlighting the restricted gene flow of SIV from blood into these organs.

**TABLE 8:** Analysis of SIV compartmentalization in male genital organs^*a*^

Macaque	Compartment	Value for:										
		Organ vs blood		Organ vs organ								
		BP	PBMC	T	EPh	EPb	EPt	VD	SV	PR	UR	LN
H1	T	**0.591**	**0.628**	0								
	EPh	0.114	**0.188**	**0.579**	0							
	EPb	**0.537**	**0.571**	0.073	**0.519**	0						
	EPt	0.075	**0.167**	**0.371**	0.111	**0.311**	0					
	VD	**0.415**	**0.473**	**0.144**	**0.426**	**0.125**	**0.241**	0				
	SV	**0.173**	**0.247**	**0.577**	**0.227**	**0.523**	**0.164**	**0.420**	0			
	PR	**0.109**	**0.143**	**0.527**	**0.130**	**0.467**	**0.102**	**0.380**	**0.093**	0		
	UR	**0.516**	**0.499**	**0.761**	**0.512**	**0.726**	**0.427**	**0.603**	**0.387**	**0.388**	0	
	LN	0.004	0.157	**0.607**	0.116	**0.541**	0.037	**0.368**	0.163	0.077	**0.506**	0
H2	EPh	**0.305**	**0.316**	NA	0							
	EPb	0.016	−0.006	NA	**0.329**	0						
	EPt	**0.348**	**0.388**	NA	**0.242**	**0.380**	0					
	VD	**0.350**	**0.387**	NA	**0.384**	**0.372**	**0.424**	0				
	SV	0.044	0.051	NA	**0.414**	0.046	**0.453**	**0.424**	0			
	PR	0.028	−0.010	NA	**0.259**	0.019	**0.316**	**0.290**	0.098	0		
	UR	**0.403**	**0.422**	NA	**0.440**	**0.425**	**0.495**	**0.465**	**0.484**	**0.382**	0	
	LN	0.025	0.024	NA	**0.289**	0.022	**0.323**	**0.323**	0.077	0.018	**0.420**	0
M1	EPh	0.056	0.051	NA	0							
	EPb	0.041	0.023	NA	0.024	0						
	EPt	0.062	**0.099**	NA	0.089	0.080	0					
	VD	0.042	0.027	NA	0.070	0.029	**0.109**	0				
	SV	0.035	0.043	NA	0.040	0.023	0.083	0.039	0			
	PR	0.022	0.018	NA	0.040	0.009	**0.108**	0.034	0.000	0		
	UR	0.114	**0.110**	NA	0.096	0.077	**0.204**	**0.105**	**0.097**	0.079	0	
	LN	0.020	0.033	NA	0.043	0.031	0.086	0.063	0.011	0.003	0.084	0

a Values in bold are significant after Bonferroni multiple test correction (H1, *P* = 0.00065; H2, *P* = 0.0007; M1, *P* = 0.0008). NA, not available because a single amplicon was recovered from testis of macaques H2 and M1.

### Male genital organs show both distinct and intermingled viral populations among themselves.

We further extended the genital organs' compartmentalization analysis to determine whether their viral populations were distinct from each other (Table 8). In macaque M1, the two genital organs that showed low levels of differentiation with blood (i.e., epididymis tail and urethra) displayed significant differences in population structuring between each other and with some other organs, albeit at very low levels. The absence of compartmentalization was confirmed by the absence of any distinct sequence distribution pattern in the phylogenetic tree or sequence motif (Fig. 6). In contrast, in macaque H2, the 4 genital organs that showed genetic differentiation with blood (epididymis head, epididymis tail, vas deferens, and urethra) displayed significant population structuring among each other, suggesting four distinct viral populations. These results are consistent with the distinct clusters/phylogenetic profile observed for these organs in the corresponding phylogenetic tree (Fig. 5). Interestingly, in macaque H1, the testis, epididymis body, and vas deferens displayed the highest differentiation from blood but showed low or nonsignificant differentiation among themselves, suggesting viral gene flow across these compartments (Table 8). This observation is further supported by the relative positions of the corresponding viral populations on the phylogenetic tree and the specific sequence motifs shared by these three tissues (Fig. 4). As a result, the genital tract of macaque H1 encompassed six compartmentalized organs potentially corresponding to four distinct viral populations. Additionally, our analysis showed high migration rates across testis, epididymis body, and vas deferens, in contrast with very low levels of incoming migration from the blood (Table 9). These results are also compatible with the spreading of a locally replicating virus strain within those tissues. Altogether, our results indicate that the male genital tract encompasses distinct viral populations within each organ, with, however, some intermingling within specific organs, such as testis and epididymis.

**TABLE 9:** Analysis of virus migration rate between testis, blood, epididymis body, and vas deferens compartments in macaque H1

Compartment into which migration occurred	Rate of migration from indicated compartment			
	T	Blood	EPb	VD
T		19.23	333.71	75.02
Blood	324.72		130.79	26.31
EPb	216.22	1.00		77.43
VD	560.18	90.82	124.39	

### Compartmentalized seminal strains arise from multiple genital organs.

We next compared semen and male genital organ viral populations to determine the source of seminal SIV. First, population structuring analysis was conducted to identify the genital organs displaying the lowest divergence with seminal plasma and semen cell viral populations (Table 10). In macaque H1, a significant differentiation was observed between semen and all genital tissues except for the seminal vesicles, pointing at this tissue as a source of virus in semen. In agreement, the phylogenetic tree comparing variants amplified from semen and all genital organs of macaque H1 showed clusters of closely related semen and seminal vesicle sequences, compatible with equilibrated virus populations, whereas sequences from all other organs and semen did not cluster together and in most cases were separated by branches with a significant bootstrap support (Fig. 4). Thus, population genetic and phylogenetic tree analyses altogether supported an equilibrium of semen and seminal vesicle virus populations, pointing to the seminal vesicles as the main source of the viruses and infected cells found in semen from macaque H1.

**TABLE 10:** Analysis of SIV compartmentalization between semen and male genital organs^*a*^

Compartment	Value for macaque					
	H1		H2		M1	
	Seminal plasma	Seminal cells	Seminal plasma	Seminal cells	Seminal plasma	Seminal cells
T	**0.564**	**0.663**	NA	NA	NA	NA
EPh	**0.243**	**0.312**	0.164	0.129	NA	**0.326**
EPb	**0.506**	**0.620**	**0.247**	**0.165**	NA	**0.343**
EPt	**0.161**	**0.251**	0.161	**0.132**	NA	**0.394**
VD	**0.404**	**0.489**	0.130	**0.198**	NA	**0.344**
SV	0.008	0.083	**0.315**	**0.232**	NA	**0.321**
**PR**	**0.084**	**0.159**	**0.163**	**0.102**	NA	**0.341**
**UR**	**0.307**	**0.357**	**0.385**	**0.360**	NA	**0.362**
**LN**	**0.171**	**0.228**	**0.188**	**0.121**	NA	**0.286**

a Values in bold are significant after Bonferroni multiple test correction (H1, *P* = 0.00065; H2, *P* = 0.0007; M1, *P* = 0.0008). NA, not available, because of small amount of amplicons testis and/or semen plasma of macaques H2 and M1.

**FIG 7:**
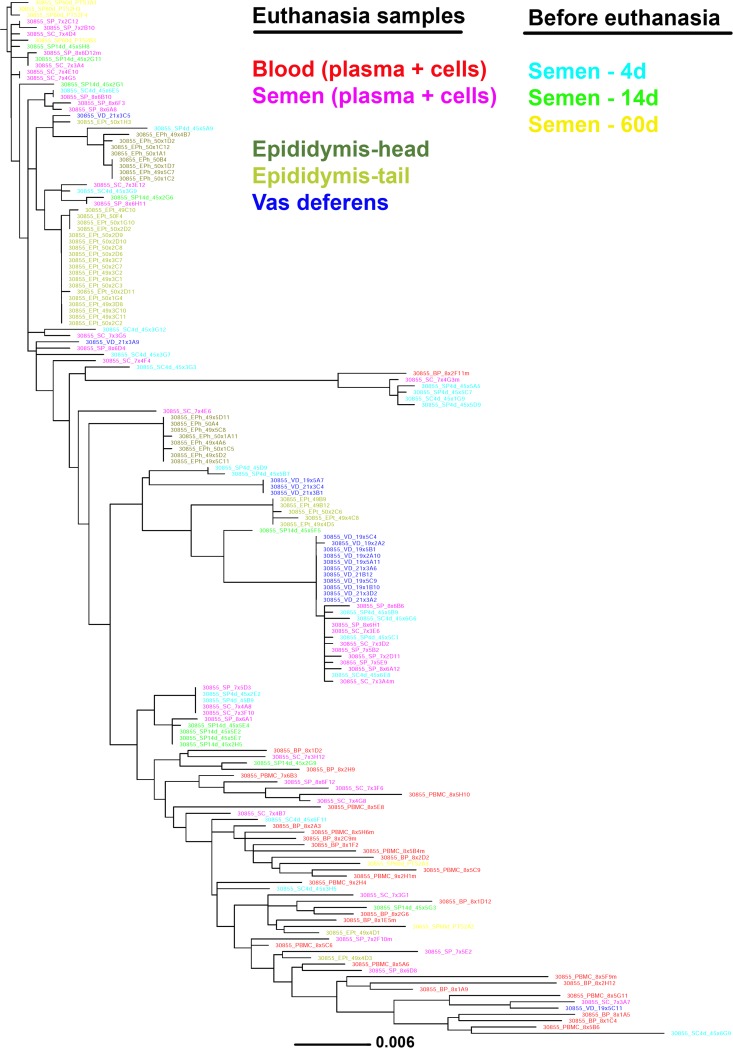
Short-term longitudinal analysis of viral populations in semen of macaque H2. Shown is a maximum likelihood phylogenetic tree generated from sequences derived from semen samples collected 4 days (blue, 22 sequences), 14 days (green, 12 sequences), and 60 days (yellow, 6 sequences) before euthanasia, together with semen (pink), blood (red), and source genital organ (vas deferens, blue; epididymis head and tail, green) sequences obtained at euthanasia. Semen samples obtained 4, 14, and 60 days before euthanasia displayed a viral population pattern similar to that of semen obtained at euthanasia, suggesting that the tissue source profile observed in a given semen sample is stable over at least several weeks rather than random. The tree was rooted using a clade (top of the tree) of the first SIV sequences collected (60 days before euthanasia) in the analysis.

In macaque H2, an absence of significant structuring was observed between seminal plasma and vas deferens, epididymis head, and epididymis tail populations. Seminal cell populations were not significantly different from epididymis head populations but differed from populations I all the other organs (Table 10); thus, a more complex pattern of genital tissue sources of seminal virus emerged for this animal. Indeed, the phylogenetic tree topology showed that the majority of semen sequences were within two distinct clusters that comprised 25% and 35% of the entire semen viral population each (Fig. 5). In the first cluster, which was supported by high bootstrap values, semen sequences were associated with nearly identical sequences from vas deferens, whereas in the second cluster, semen sequences were associated with sequences from epididymis head and tail (Fig. 5). Of note, Highlighter plots revealed that colocalization of organ and semen sequences was mostly driven by the presence of the same specific sequence motif (Fig. 5). These results suggest that in macaque H2, the virus present in semen had a multitissue origin, i.e., vas deferens and distinct anatomical parts of the epididymis. Interestingly, a longitudinal analysis of semen samples collected at 4, 14, and 60 days before euthanasia of animal H2 showed that the tissue sources of seminal virus remained stable over several weeks (Fig. 7).

In macaque M1, high levels of viral population differentiation were observed between seminal cells and genital organs, and therefore, none of the tissues analyzed could be identified as the source of infected cells (Table 10). Phylogenetic analysis confirmed this result, since none of the genital organ sequences could be found in the two distinct clusters of semen sequences (Fig. 6). The failure to identify local sources in this animal may be due to the difficulty in obtaining a sufficiently high number of sequences from its genital organs. Overall, our results indicate that several genital organs can release free virus and infected cells into semen and that the sources of virus in semen vary among individuals.

**FIG 8:**
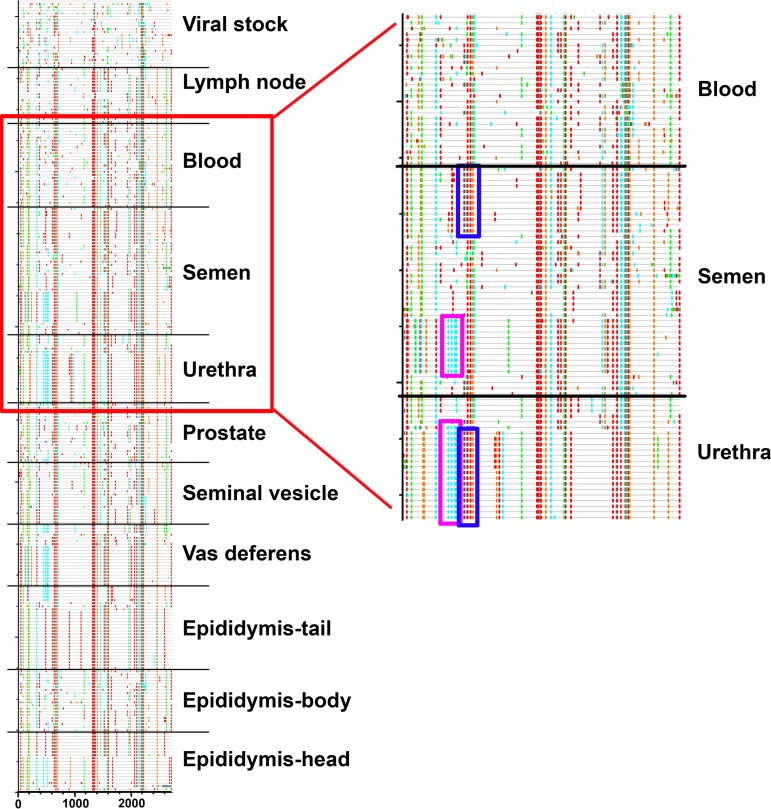
Comparison of semen and urethra sequence motifs in macaque H2. (Left) Highlighter plots generated from all sequences derived from macaque H2 together with viral stock. Sequences are grouped by tissue. (Right) Detailed view of the area indicated on the left within the red rectangle. The two 150-bp motifs which are absent from any blood related sequences are observed either separated in semen-related sequences or fused in urethra sequences. This pattern may result from recombination events between semen sequences after infection of the urethra.

Interestingly, whereas phylogenetic and population structuring results were not compatible with the urethra as a source of virus in semen, the short distance between semen and urethra sequence clusters in the phylogenetic tree of animal H1, together with the presence of specific sequence motifs in those 2 virus populations, suggested a link between these compartments (Fig. 4). The same observation applied to animal H2: a specific motif of 300 bp within urethra viral populations corresponded to the fusion of two separated motifs around 150 bp each that were highly abundant within seminal strains and their source organs (Fig. 8), suggesting a recombination event. In agreement, migration analysis revealed more migrants flowing from seminal cells and seminal plasma into the urethra than the other way round (Table 11). Together, these observations suggest that semen virus may reinfect the urethra, bringing semen-specific features within the urethra viral population.

**TABLE 11:** Analysis of virus migration rate between seminal cells, seminal plasma, and urethra in macaques H2 and H1^*a*^

Macaque	Compartment into which migration occurred	Rate of migration from indicated compartment		
		SC	SP	UR
H2	SC		1,446.40	266.62
	SP	375.69		199.30
	UR	617.66	559.32	
H1	SC		1,288.91	296.86
	SP	1,318.59		207.96
	UR	672.72	662.60	

a SC, seminal cells; SP, seminal plasma; UR, urethra.

## DISCUSSION

We aimed to identify the sources of the AIDS virus in semen. To this end, we first examined the infection level and inflammation status of all of the 4 organs (testis, epididymis, seminal vesicles, and prostate) involved in semen elaboration, as well as of 2 ducts involved in semen transport (vas deferens and urethra) from 8 chronically infected macaques. Numerous studies reported a significant correlation between HIV shedding and elevated cytokine concentrations in semen, along with specific pattern of cytokine network in semen compared with blood, suggesting that inflammation favors the burst/release of virus from local sources (17–21, 25–30). Our data showed no correlation between semen viral load and expression levels of proinflammatory cytokines such as TNF-α and IL-1β in infected semen-producing organs, preventing initial discrimination of potential tissue sources.

We next compared the genetic diversity and structure of viral populations in male organs, as well as in semen and blood cells and plasma for three animals with compartmentalized semen strains. Our data revealed that a number of male genital organs harbor viral strains phylogenically distinct from those in blood and other genital organs, resulting in the presence of multiple distinct viral populations within the male genital tract. These results were then confirmed by our compartmentalization analyses (Tables 8 and 10). These findings are in contrast with those of Fieni et al., who failed to observe compartmentalized strains in testis, epididymis, seminal vesicles, prostate, and semen from 5 rhesus macaques infected with SIVmac251 for 3 months (4). Differences in experimental procedure, such as duration of infection, male genital organs analyzed (vas deferens and urethra were not included in that study and the section of epididymis screened was not specified), and inoculation mode (penile versus intravenous inoculation), together with interindividual differences may explain this apparent discrepancy.

Our analyses were performed in macaques infected by the intravenous route. This route of infection differs from the most common route of HIV acquisition in men, i.e., sexual transmission. Men can sexually acquire HIV through either the rectal or penile mucosa (31–38). During sexual transmission, only one or very few viruses establish the infection in the new host, despite the high diversity of HIV-1 populations in the blood and genital secretions of the donor (39). Interestingly, such a bottleneck has also been reported to occur for transmissions in intravenous drug users, with about 60% of individuals infected with a single variant (40). In agreement with the latter, our population dynamic analysis, including all the macaques and inoculum sequences suggested one single origin for each macaque infection (one most recent common ancestor [MRCA]). Differences in selection pressures among the different transmission modes can influence the genetic composition of the founder virus, as shown for heterosexual versus homosexual transmission (40). Thus, the infection mode (e.g., intravenous versus penile) likely shapes the initial selection of the transmitted virus. In addition, the timing and mechanism of viral dissemination may differ for specific organs, such as the urethra, which can constitute a portal of entry for HIV during penile infection, in addition to foreskin (31). However, the viral seeding of male genital organs is considered to predominantly occur during peak viremia following spillover from blood, in which much higher viral load and infected cell number are encountered than in the male genital tract (41). In agreement, infection levels in testis, epididymis, prostate and seminal vesicles of macaques infected by penile inoculation correlated with blood infection levels in the early phase of infection (11 weeks postinfection) (4), similar to the case with animals intravenously infected (9). Compartmentalization in the male genital tract presumably occurs because of restricted gene flow between blood and male organs, after host control sharply decreases viremia, leading to localized diversification of variants in the male tissues. The lack of a specific semen signature in semen variants from SIV-infected macaques in the early infection stage further suggests that there is no determinant of viral tropism in the male genital tract (4, 41). The absence of viral compartmentalization in semen during the early stage of infection in macaques infected by the intrapenile or intravenous route further suggests that compartmentalization and seminal shedding are independent of the infection route (4, 41). Moreover, because in our study the animals were well into the chronic stage (at a minimum of 6 months' distance from the initial seeding stage), it is unlikely that the infection route influenced seminal shedding or viral compartmentalization.

**FIG 9:**
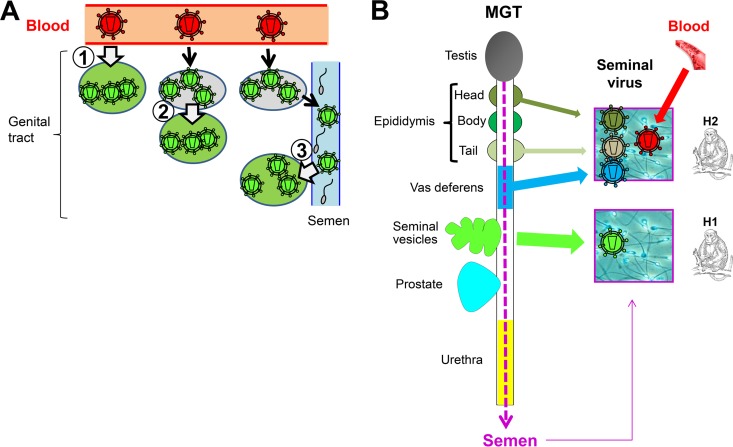
Origin of virus in male genital organs and semen. (A) Model for virus seeding in male genital organs: virus in male genital organs may originate from blood (1), from another genital organ as a result of close vicinity or connected vasculature system (as observed between vas deferens, epididymis body, and testis in macaque H1) (2), or from a distant genital organ by transmission via semen, as suggested for urethra in macaque H1 and H2 (3). (B) Scheme illustrating the male genital organs releasing virus in semen for 2 macaques with compartmentalized strains in semen.

Viral populations in all genital organs and semen were phylogenetically very distinct from the viral inoculum, suggesting that the analysis of animals 6 to 15 months after the injection allowed for tissue-specific replication and selection. No semen- or organ-specific signature was identified in our study, which is not surprising considering the small number of animals analyzed and the fact that, except for one study (42), such a signature has not been evidenced in semen from HIV^+^ men (24, 43) or SIV+ macaques (41). Interestingly, we found that most of the macaques with highly compartmentalized SIV populations displayed very low genetic diversity and clusters of nearly identical sequences on the phylogenetic tree. This is unlikely to result from sampling bias since several tissue fragments were used and because this low diversity occurred irrespective of infection level. Similar clustering of nearly identical viral sequences and loss of genetic diversity were described for semen from HIV^+^ men and postulated to result from the clonal amplification of infected cells within the genital tract (24). The current hypothesis for the infection of body organs distant from the entry point of the transmitted virus is an initial seeding with blood-borne virus during the widespread early stage of the infection, followed by a stage with restricted gene flow and viral replication under local immune selective pressure specific for the organ once peak viremia is reduced. Alternatively, the low genetic diversity combined with the relatively low level of infection in genital organs might suggest initial colonization of the male genital organs by a small number of variants from a large blood population (founder effect). In favor of the first hypothesis, however, we previously reported higher infection levels and higher number of infected cells in situ in genital organs from acutely infected macaques than in chronically infected macaques (9). The results from Whitney et al. showing compartmentalized virus in semen of chronically but not acutely infected macaques (41) also support this first model. Interestingly, our data suggest two additional ways of virus seeding in male genital organs, both resulting from intercompartment infection (Fig. 9A). First, we observed a strong convergence of viral populations from the testis, the body of the epididymis, and the vas deferens from macaque H1, compatible with the spreading of a locally replicating virus strain within these tissues, in close spatial vicinity and with interconnected vasculature and drainage. Second, although the viral populations in semen and urethra were clearly distinct, we observed striking common features between those populations in macaques H1 and H2. Migration and compartmentalization analyses suggested viral migration from semen to the urethra. Because residual semen can remain within the urethral lumen, it is physiologically possible that semen strains contaminated the urethra following ejaculation, thus mixing with the strains already present in this tissue in a unique manner. The urethra is indeed a well-identified portal of entry for various pathogens present in the penile lumen, including HIV (31). This transmission mode could constitute another intercompartment infection, here mediated by semen.

Importantly, our study is the first to identify several genital organs with viral sequences similar to those in viral particles and infected cells present in semen. Semen is composed of seminal plasma (which arises from the secretions of the seminal vesicles and prostate and, to a lower extent, from the epididymis) and of a cell fraction containing primarily spermatozoa (produced in the testis before being matured and stored in the epididymis), together with leukocytes of undetermined origin. During ejaculation, the sperm is transported from the epididymis through the vas deferens ducts to the seminal vesicles, where it combines with its secretions, and then from the seminal vesicles through the ejaculatory ducts to the prostate, to finally travel through the urethra before its release through the meatus (urethra opening). We demonstrated that 100% of the semen viral quasispecies match viral populations from the seminal vesicles in macaque H1, whereas in macaque H2, around 60% of semen strains match viral populations from the epididymis and the vas deferens (Fig. 9B). Early studies investigated the sources of HIV in semen by comparing HIV RNA levels in semen versus urethral swabs or prostatic massages, or before and after vasectomy in therapy-naive HIV^+^ men (2, 44). They concluded that seminal HIV arose distant from the testis and epididymis, suggesting the prostate and/or urethra as a key player. However, the lack of information on the compartmentalization status of the viral strains in semen was a major confounding factor of those studies solely relying on viral loads. Thus, semen viral populations in the individual tested may have arisen exclusively from blood, as we evidenced here for a subset of animals and as previously described for men (1). Consistent with our results, a recent study found that the urethra was not the source of persistent viral RNA in semen from HIV-infected men under suppressive highly active antiretroviral therapy (HAART) (45). Thus, although penile urethra displayed the highest level of infection of all male genital organs after 4 months of HAART in macaques (14), other local sources may be responsible for the release of HIV RNA in semen during suppressive HAART. Our observation that the epididymis represents an important source of infected cells in semen is in agreement with vasectomy leading to reduced seminal leukocyte numbers in healthy men (46), along with the localization of infected leukocytes near and inside the epididymal lumen in men and macaques (9, 12, 13). The excretion of viruses and infected cells from the seminal vesicles, an organ so far largely ignored when considering potential sources of HIV in semen, is compatible with the localization of infected cells close to its secretory epithelium and within its tubular lumen in infected men and macaques (7, 9, 12), along with the fact that the seminal vesicles produce approximately 60% of the seminal fluid. Collectively, our findings indicate that the release of viruses in semen is a complex individual-dependent process, involving the genital glands, which produce the seminal plasma (e.g., seminal vesicles), as well as organs and ducts through which semen travels (e.g., epididymis and vas deferens). It is possible that the analysis of a larger number of animals would have revealed other genital organs involved in virus shedding. Interestingly, the individual-specific pattern of seminal excretion (i.e., from distinct organs) was irrespective of genital organs' SIV infection or compartmentalized levels. Whether the animal-specific origin of seminal sequences results from virus- or host-specific characteristics requires further investigation.

When comparing RNA sequences of viruses produced by cells and tissues with RNA from the viral particles released in semen and blood, we did not observe any difference between cell-free and cell-associated semen virus populations, indicating common sources. Whereas differences between the cell-free and cell-associated virus have been reported (47, 48), our results are in agreement with recent analyses (49–52) demonstrating a common source for cell-free and cell-associated semen virus. Differences in starting materials (DNA versus RNA) as well as in amplification methods (bulk PCR or single-genome amplification), amplicon sizes, and genome areas may account for this discrepancy.

Compartmentalization in semen has been reported to be a transient phenomenon, with variations sometimes as short as within few days (53). This could reflect a very versatile compartmentalization status of the local source(s) and/or changes in the origin of seminal virus between ejaculates. Our finding that several semen-producing organs can potentially represent a source of compartmentalized virus is compatible with fluctuations of the seeding sources over time. While longitudinal follow-up of viral populations in genital organs is technically not possible, the high level of compartmentalization and low genetic diversity observed in several genital tissues (e.g., urethra and vas deferens) suggest medium to long-term selection and relative stability over time. Such relative stability was evidenced by our longitudinal semen analysis, which revealed that compartmentalized seminal viral populations originated from the same source over a 2-month period. Consistent with our results, Gupta et al. found the same pattern of compartmentalization in semen for over 10 weeks and associated compartmentalization with intermittent HIV shedding in semen (54). This suggests that the transient nature of compartmentalized strains in semen reported for HIV^+^ men over relatively short periods is likely to reflect the natural variation of semen composition rather than an extreme instability of the compartmentalization status of genital organs.

This study is the first to identify specific male organs that release SIV into semen and to uncover the viral populations present in male genital tissues. Deciphering the origin of viruses in semen is crucial to improve our understanding of the persistence of HIV and other emerging viruses in this body fluid. We showed that rather than a single source, a number of genital organs are involved and that the nature of the shedding male organs varies among individuals. Importantly, we found that previously overlooked genital organs, such as the seminal vesicles and the epididymis, release both infected cells and free virions into semen. These results pave the way for further studies to determine the viral and host-specific characteristics associated with male organs' compartmentalization and virus excretion into semen.

## MATERIALS AND METHODS

### Ethics statement.

Adult cynomolgus macaques (Macaca fascicularis) imported from Mauritius were housed in the facilities of the Commissariat à l'Energie Atomique et aux Energies Alternatives (CEA; Fontenay-aux-Roses, France). Nonhuman primates (NHP) are used at the CEA in accordance with French national regulations (CEA permit number A 92-032-02). The CEA is also in compliance with Standards for Human Care and Use of Laboratory of the Office for Laboratory Animal Welfare (OLAW, USA) (55) under OLAW assurance number A5826-01. The use of NHP at CEA is in accordance with the recommendation of the European Directive (2010/63/CE). Animals were housed in adjoining individual cages allowing social interactions, under controlled conditions of humidity, temperature, and light (12-h light/12-h dark cycles). Water was available ad libitum. Animals were monitored and fed 1 or 2 times daily with commercial monkey chow and fruits by trained personnel. Macaques were provided with environmental enrichment, including toys, novel foodstuffs, and music, under the supervision of the CEA Animal Welfare Body. The protocols employed were approved under statement number 10-060 (13 November 2012) by the ethics committee of the CEA Comité d'Ethique en Expérimentation Animale, registered by the French Research Ministry under statement number 44. The animals were used under the supervision of the veterinarians in charge of the animal facility. Experimental procedures were conducted after animal sedation with ketamine chlorydrate (Rhone-Merieux, Lyon, France; 10 mg/kg of body weight) as previously described (55). Tissues from the MGT were collected during animal necropsy at the end of the treatment after sedation of animals (ketamine chlorhydrate at 10 mg/kg) followed by euthanasia (sodium pentobarbital at 180 mg/kg). It should be stressed that none of the animal were specifically used for this work, since semen, blood, lymph nodes, and male genital organs were collected in the course of previous studies from our laboratory (14, 56); thus, no suffering was specifically associated with the procedure to collect these samples. This approach is fully in accordance with the principles of the 3Rs (replacement, reduction, and refinement) and reduces the number of animals used as recommended by directive 2010/63/CE (article 18, on sharing tissues and organs).

### Study animals and tissue collection.

The 8 macaques selected for this study were intravenously inoculated with 50 50% animal infectious doses (AID_50_) of pathogenic cell-free SIVmac251 in 1 ml of phosphate-buffered saline (PBS). Of note is that one of the macaques (H1; 21362) was in the AIDS stage. The generation and titration of the SIVmac251 virus stock have been described elsewhere (55). Semen, blood, genital tract tissues (testis, three morphologically and functionally distinct regions of the epididymis [head, body, and tail], vas deferens, seminal vesicles, prostate, and urethra) and inguinal lymph nodes (included as a local yet non-male-genital organ) were collected at the time of euthanasia, 6 to 15 months postinfection. The semen and blood samples were separated into plasma and cell fractions by centrifugation and stored in −80°C. MGT tissues were cut into fragments (around 300 mg each) and stored −80°C or fixed in 4% formaldehyde.

### Quantitative SIV RNA analysis.

Blood and plasma viral loads were determined as previously described (55). For SIV RNA quantification in tissues, total RNA was extracted from frozen tissues using the RNeasy isolation kit (Qiagen) according to the manufacturer's protocol. RNA samples were DNase treated (Promega) and reverse transcribed into cDNA using the high-capacity RNA-to-cDNA kit (Applied Biosystems) following the manufacturer's recommendations. Synthesized cDNA was subjected to quantitative SIV RNA analysis using a preamplification step followed by a quantitative real-time (TaqMan) PCR step as described previously (14).

### Single-genome amplification.

In order to maximize the chances of detecting viral sequences in cells and organs matching those of free virus in semen, we chose to focus on the viral strains being currently produced and therefore used viral RNA in tissues and cells for sequencing rather than viral DNA, which can represent archived/defective viruses. Given that both cell-associated and cell-free viruses are present in semen, and that it is currently not clear which of these initiates a new infection, we chose to amplify both cell-associated and free virus in semen. Total RNA was isolated from blood and semen plasma and infected cells using TRIzol-LS and TRIzol, respectively, according to the manufacturer's instructions (Invitrogen). Genital tract tissue samples were homogenized with a TissuLyser II system (Qiagen), and total RNA was extracted using TRIzol (Invitrogen) following the manufacturer's suggested protocol. RNA samples were DNase treated with RQ1 RNase-Free DNase (Promega) for 30 min at 37°C and reverse transcribed into cDNA as previously described (34), with minor modifications. Briefly, RNA, deoxynucleoside triphosphates (1 mM), and 1 μM primer EnvR1 (5′-TGTAATAAATCCCTTCCAGTCCCCCC-3′) were incubated for 6 min at 65°C, followed by incubation at 4°C for 3 min. Then a mix of the following was added: 4 μl of 5× first-strand buffer, 1 μl of 100 mM dithiothreitol, 0.5 μl of RNaseOUT recombinant RNase inhibitor (40 U/μl; Invitrogen Life Technologies), and 1 μl of SuperScript III reverse transcriptase (200 U/μl; Invitrogen Life Technologies). The reaction mixture was then incubated at 53°C for 60 min, followed by 15 min at 70°C. cDNA was stored at −80°C until further analysis. Full-length 3.2-kb SIV *env* gene amplicons were obtained using single-genome amplification as previously described (34, 57), with minor modifications. The optimal SGA working dilution was determined for each cDNA sample using 4-fold serial dilutions (ranging from 1/4 to 1/256 for all samples except for inguinal lymph node tissue samples, 1/4 to 1/4,096) in double-distilled water distributed in replicates of 6 PCRs. According to the Poisson distribution, the DNA dilution yielding positive PCR products in no more than 30% of the wells will provide amplicons resulting from single molecule amplification in over 80% of these wells. The corresponding dilution, designed as SGA working dilution, was used to perform additional PCR amplifications. SGA PCR conditions were as follows: the first-round PCR was performed using 1 μl of cDNA dilution in a 96-well plate with 1× Phusion HF buffer, 0.2 mM each deoxynucleoside triphosphate, 0.3 μM primers SIVsm/macEnvF1 (5′-CCTCCCCCTCCAGGACTAGC-3′) and SIVsm/macEnvR1 (5′-TGTAATAAATCCCTTCCAGTCCCCCC-3′), and 0.016 U/μl of Phusion Hot Start Flex DNA polymerase (New England BioLabs) in a 25-μl reaction volume. The following cycling conditions were used: 98°C for 45 s followed by 35 cycles of 98°C for 15 s, 55°C for 30 s, and 72°C for 6 min and a final extension of 72°C for 10 min. The second-round PCR was carried out using 1 μl of the first-round product with primers SIVmacEnvF2 (5′-TATAATAGACATGGAGACACCCTTGAGGGAGC-3′) and SIVsmEnvR2 (5′-ATGAGACATRTCTATTGCCAATTTGTA-3′) and the same PCR mixture as in the first round. The cycling conditions were 98°C for 45 s followed by 40 cycles of 98°C for 15 s, 55°C for 30 s, and 72°C for 5 min and a final extension of 72°C for 10 min. Amplicons were detected (or inspected) using the long amplicon melt profiling (LAMP) method described previously (16). Briefly, the melting analysis was carried out in a 96-well plate with 1.2 μl of second-round PCR product, 2 μl of IQ SYBR green supermix, and 0.16 μl of ROX passive reference dye (Bio-Rad) in an 8-μl reaction mixture using a 7500 real-time PCR system instrument (Applied Biosystems). The following melt curve run method was used: 95°C for 15 s and 60°C for 1 min, followed by an increase to 95°C with a ramp rate of 1% and continuous data acquisition. Positive reactions were isolated based on the specific melting profile of the full-length *env* amplicon. PCR products were directly sequenced on both strands as partially overlapping fragments by Eurofins Company. Individual sequence fragments for each amplicon were manually examined for multiple peaks (to exclude sequences arising from multiple variant templates) and assembled using the Contig Editor program from GeneStudio.

### Sequence alignment and phylogenetic reconstruction.

A total of 708 full-length SIV *env* sequences of semen and blood from 5 animals and genital tract tissues from 3 animals were selected (24 to 187 sequences per macaque). Nucleotide sequences were translated into amino acids and aligned in MAFFT (58) using the global algorithm (G-INS-i). Alignments were visually inspected and adjusted if needed and then translated back to nucleotides. Strains containing internal stop codons were counted and removed. The best-fit model of DNA substitution was selected with the Akaike information criterion as implemented in jModelTest (59). Maximum likelihood phylogenetic trees were inferred in RAxML (60) and rooted using the viral inoculum sequences as the outgroup with the exception of the tree in Fig. 7 (longitudinal analysis of semen sequences), which was rooted using a clade of the first SIV sequences collected (60 days before euthanasia) in the analysis. Nodes with bootstrap values of ≥70% (1,000 bootstrap replicates) are labeled. Heuristic searches were performed under the best-fit model. Bayesian trees were also inferred using MrBayes (61). We ran four chains (one cold and three heated) for 10^7^ generations, sampling every 1,000 steps. Each run was repeated twice. Convergence and mixing of the Markov chains were assessed in Tracer (62).

### Population inference.

Genetic diversity (θ) (63) and population recombination rate (ρ) were estimated for each macaque using DNAsp and LDhat (64), respectively. Migration rates (i.e., rates at which migrants enter a population) were estimated using the ML coalescent approach implemented in LAMARC (65), while accounting for recombination and population growth. Molecular adaptation was assessed using the ratio of nonsynonymous (*dN*) to synonymous (*dS*) substitution rates (ω) and estimated using the model M0 (one ratio) and the mixed-effects model of evolution (MEME) in HyPhy (66). Recombination was taken into account by first detecting recombination breakpoints with a genetic algorithm for recombination detection (GARD) and then estimating the *dN*/*dS* ratios independently for each fragment. Population dynamics were inferred in BEAST (67) using the Gaussian Markov random field (GMRF) skyride model, the HKY substitution model with gamma-distributed among-site rate heterogeneity, and a relaxed clock (log-normal) model of rate of substitution. We used the date of infection to calibrate the analysis. We performed two runs 2 × 10^7^ generations long. Intrahost phylogeographic history (viral dispersal patterns and divergence times), while accommodating phylogenetic uncertainty, was inferred between body compartments using asymmetric discrete phylogeographic diffusion models (68). Results were summarized with a maximum clade credibility (MCC) tree, using TreeAnnotator. The MCC tree was visualized with FigTree (http://tree.bio.ed.ac.uk/software/figtree). Parameter uncertainty was summarized in the 95% highest posterior density (HPD) intervals. All output generated by BEAST was analyzed in Tracer (http://tree.bio.ed.ac.uk/software/tracer/) to test for convergence and mixing.

### Compartmentalization detection.

Compartmentalization among body compartments in each macaque was assessed using several approaches, including distance-based (*F*_ST_ and nearest neighbor [*S*_nn_]) and tree-based (Slatkin-Maddison [SM], association index [AI], correlation coefficients [*r* and *r_b_*]) estimators implemented in HyPhy (see references therein). Viral populations were considered compartmentalized when all tests converged.

### Statistical analysis.

Statistical analyses were performed using commercially available software GraphPad Prism version 5.03. Spearman's rank correlation test was used to assess nonparametric associations between virus and cytokine RNA concentrations. All concentration variables were log_10_ transformed. Significance of distance-based values was tested using nonparametric permutation procedures (10,000 permutations) (69). When multiple comparisons were made, *P* value threshold for statistical significance was adjusted using the Bonferroni correction.
